# Are videos uploaded by dental professionals on lip repositioning surgery of higher quality? A youtube video analysis

**DOI:** 10.1371/journal.pone.0327194

**Published:** 2025-09-19

**Authors:** Ebru Ece Sarıbas, Muzeyyen Kandemır

**Affiliations:** 1 Dicle University Faculty of Dentistry, Department of Periodontology Diyarbakır, Turkey; 2 Dicle University Faculty of Dentistry, Department of Periodontology Diyarbakır, Turkey; Ege University, Faculty of Medicine, TÜRKIYE

## Abstract

Lip repositioning surgery is a minimally invasive procedure used in the treatment of gummy smile. With the increasing demand for aesthetic dental procedures, platforms like YouTube™ have become popular sources for visual health information. This study aimed to evaluate the quality, reliability, and educational value of YouTube™ videos related to lip repositioning surgery and to identify factors influencing video quality. This research was conducted on YouTube™ using the term “lip repositioning” on February 20, 2025. The first 150 videos sorted by relevance were screened. According to the inclusion criteria, 53 videos were recorded. Data such as video duration, upload source, view count, comments, likes/dislikes, and country of origin were recorded. Viewer engagement was analyzed through interaction index and viewing rate. Content was evaluated using the Video Content Quality (VCQ) score, Global Quality Scale (GQS) and DISCERN tool. Statistical analyses were performed with a significance level of *P* < 0.05. Most videos were uploaded by dentists (64.2%), and 71.7% were educational. The mean VCQ score was 9.98 ± 3.95, indicating low-to-moderate content quality. Videos uploaded by professionals had higher quality scores (p < 0.001), while 56.6% were of poor content quality. Although YouTube™ is a widely used source for health information, videos on lip repositioning surgery lack sufficient educational value and reliability. Professional content creation should be encouraged.

## Introduction

A smile is a universal form of expression that plays a significant role in an individual’s self-confidence and social interactions. [1]. The “ideal smile” refers to a situation where the crowns of the maxillary teeth are fully visible, along with 1–2 mm of gingival display. When 2 mm or more of gingiva is visible during smiling, it is referred to as a “gummy smile.” This condition is observed in approximately 10.5% to 29% of adults and is more commonly seen in women [2].

Multiple etiological factors contribute to gummy smile, including altered passive eruption, dentoalveolar extrusion, vertical maxillary excess, or hyperactive upper lip musculature. [3]. In order to determine the appropriate treatment option, it is essential to first identify the underlying etiological factor accurately.

Lip repositioning surgery, first described by Rubinstein and Kostianovsky in 1973, is a minimally invasive treatment that restricts the elevation of the upper lip, offering a conservative and predictable solution with high patient acceptance. [4].

With the advancement and widespread use of technology, internet access has significantly increased. In a study conducted in Europe with 7,934 participants, it was reported that 71% of the participants were internet users, and 44% of them used the internet specifically to obtain health-related information [5].

With the rise of internet use, YouTube™ has become a prominent source of health-related information. [6]. While numerous medical studies have assessed the quality of health-related content on YouTube™, similar research remains scarce within the field of dentistry. To date, there appears to be no published study specifically evaluating the informational quality of YouTube™ videos concerning lip repositioning surgery.

Therefore, this study aimed to evaluate the quality, reliability, and educational value of YouTube™ videos related to lip repositioning surgery. We hypothesized that videos uploaded by dental professionals would demonstrate higher quality and reliability compared to those from other sources.

## Materials and methods

The present study was conducted over a one-month period from March 15 to April 5, 2025. As all analyzed videos were publicly accessible, ethical approval was deemed unnecessary. This study utilized publicly accessible data from YouTube™ and Google Trends. All procedures for data collection and analysis were conducted in accordance with the platforms’ terms of service and do not violate user privacy or content policies.

**Fig 1:**
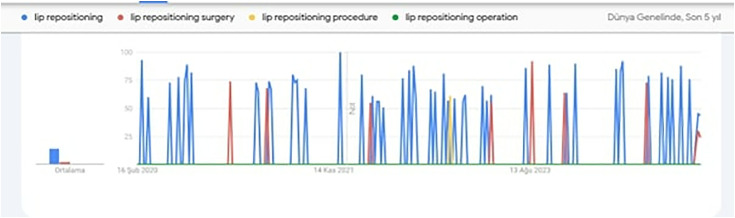
Results of the google trends keyword search.

To identify the most commonly searched term related to our topic, the “Google Trends” tool was used. Google Trends is an online search tool that organizes the most frequently used keywords, topics, and expressions within a specific timeframe and across various regions of the world. In the Google Trends application, the search settings were adjusted to “worldwide” and “past 5 years,” and potential keywords such as “lip repositioning,” “lip repositioning surgery,” and “lip repositioning operation” were compared. It was observed that “lip repositioning” was the most frequently searched term (Fig 1).

After determining the keyword, Google Chrome’s incognito mode was activated and a new YouTube™ user account was created to ensure that the collected data would not be influenced by previous search history or saved videos, thus maintaining objectivity. The search was conducted on February 20, 2025, using the term “lip repositioning.” Previous studies have shown that while YouTube™ users may browse through anywhere between 60–200 videos when searching for information, they typically view only the first 30 videos [7,8]. Moreover, it has been reported that the vast majority of users access only the first three pages of a search—approximately the first 120 videos [8]. Based on these findings, our study included only the first 150 videos to ensure consistency with prior research and relevance to the evaluation criteria. No additional filtering options were deliberately selected; instead, only YouTube’s default “sort by relevance” setting was used. This approach was chosen to align the search process with common user behavior.

**Fig 2:**
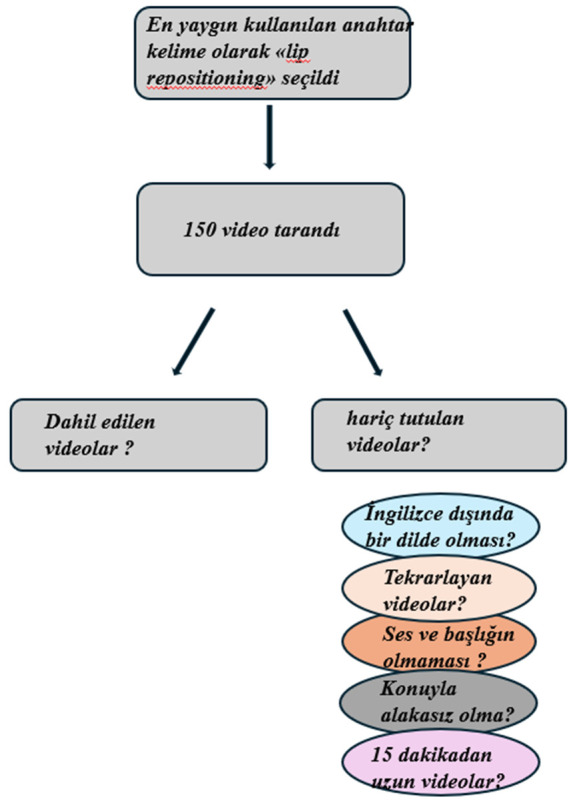
Video selection criteria.

All of the initially recorded 150 videos were reviewed, and based on the exclusion criteria, 97 videos were excluded from the study, while 53 videos were included (S1 Table). Exclusion criteria are given in Fig 2.

The 53 videos included in the study were independently reviewed by two researchers: E.S, an assistant professor in the Department of Periodontology, and M.Ö, a research assistant. For each video, demographic information such as upload date, source, duration (in minutes), number of comments, number of views, likes and dislikes and country of origin was recorded [9]. To ensure the researchers’ objectivity, the number of likes and comments was not viewed until after the video had been fully watched.

Based on the source, videos were classified into three categories: dentist/specialist, advertising companies, and individual users. According to their content, videos were also categorized as patient experience, educational videos, and scientifically inaccurate. Based on the data obtained, two quantitative measures were used to assess viewer engagement: the interaction index and the viewing rate. The interaction index was calculated by subtracting the number of dislikes from the number of likes, dividing the result by the total number of views, and then multiplying by 100. Similarly, the viewing rate was computed by dividing the total number of views by the number of days since the video was uploaded and multiplying the result by 100 [10].

**Fig 3:**
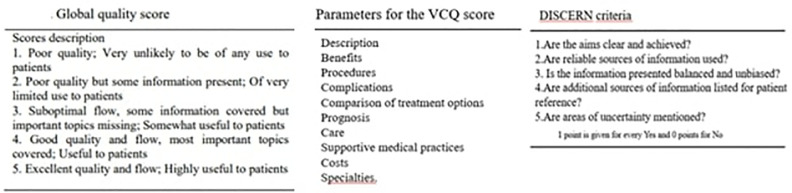
GQS score, VCQ score and DISCERN questionnaire items.

To obtain an objective evaluation of each video’s content, the Video Content Quality (VCQ) score and the Global Quality Scale (GQS) score were recorded [11,12] (S2 Table). The GQS is a 5-point Likert scale that assesses a video’s overall quality, including the quality of information, logical flow, and ease of use. Scores range from very poor quality (1 point), for poor quality with limited usefulness (2 points), for average quality (3 points), for good quality (4 points) and for excellent quality (5 points). The VCQ score was based on 10 content-related parameters, each rated on a scale from 0 to 3. The total score ranged from 0 to 30 [6] (Fig 3). Finally, the reliability of all included videos was evaluated using a five-question survey adapted from the DISCERN tool, which is commonly used to assess the quality of written consumer health information (Fig 3) [13]. DISCERN scoring was conducted using a five-question survey adapted from the original DISCERN tool, which is widely used to assess the quality of consumer health information. Each question was scored as follows: 1 point is given for every Yes and 0 points for No. The total DISCERN score ranged from 0 to 5, with higher scores indicating greater reliability and quality.

### Statistical analysis

The distribution of continuous variables was evaluated using the Shapiro-Wilk test to determine normality. Variables conforming to a normal distribution were summarized using mean ± standard deviation, while those not meeting the assumption were described using median (minimum–maximum) values. Categorical variables were presented as frequencies and percentages [n (%)]. For comparisons involving two independent groups, the Mann–Whitney U test was applied when the assumption of normality was not satisfied. In analyses with more than two groups, the Kruskal–Wallis test was used. Where statistically significant differences were observed, post hoc evaluations were conducted using the Dunn–Bonferroni test. The relationships between VCQ, GQS and DISCERN scores according to the duration of Youtube videos were examined by correlation analysis and Spearman correlation coefficient was calculated. For statistical analyses, SPSS (IBM Corp. Released 2017. IBM SPSS Statistics for Windows, Version 25.0. Armonk, NY: IBM Corp.) programme was used and type I error rate was accepted as 5%.

## Results

In our study, all 150 initially recorded videos were reviewed, and based on the exclusion criteria, 97 videos were excluded while 53 videos were included in the analysis.

The descriptive characteristics of YouTube™ videos related to lip repositioning surgery were examined. The mean duration of the videos was approximately 3.93 ± 3.32 minutes, the total number of days since the videos were uploaded had a mean of 2009.53 ± 1412.8 days.

**Fig 4:**
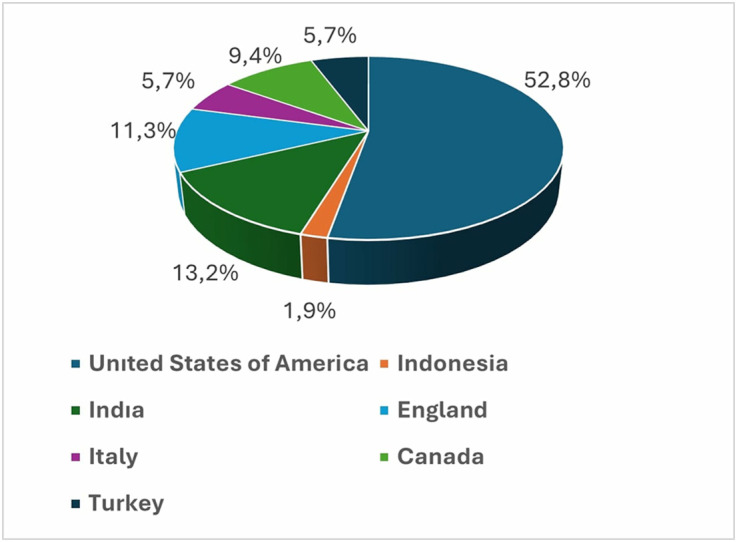
Distribution of countries.

The mean interaction index was 0.99 ± 0.83, the mean viewing rate was 1021.46 ± 2518.07. The number of views had a mean of 20,088.02 ± 58,760.54. The mean number of likes was 198.81 ± 617.36 and the mean number of comments was 20.25 ± 46.11. The mean VCQ score was found to be 9.98 ± 3.95, the mean GQS score was 2.91 ± 0.99, and the mean DISCERN score was 2.77 ± 1.09. Regarding the source of the videos, 64.2% were uploaded by dentists. In terms of countries, the majority of the videos (52.8%) were uploaded from the United States (Fig 4). When examining the types of video content, 71.7% were educational videos (Table 1).

**Table 1:** Descriptive characteristics of YouTube videos.

	Mean	SD	Medyan	Minimum	Maximum
**Video Duration (min)**	3,93	3,32	2,43	1	14,32
**Total Number of Days Since Upload**	2009,53	1412,8	1727	190	5096
Interaction Index	0,99	0,83	0,72	0	3,73
Viewing Rate	1021,46	2518,07	144,4	6,43	12353,74
Number of Views	20088,02	58760,54	2471,5	41	351065
Number of Comments	20,25	46,11	5	0	238
Number of Likes	198,81	617,36	12	0	3500
**VCQ Score**	9,98	3,95	9	2	23
**GQS Score**	2,91	0,99	3	1	5
**DISCERN Score**	2,77	1,09	3	0	5
	**N**		**%**		
**Video Source**					
Individual User	15		28,3		
Dentist	34		64,2		
Advertisement	4		7,5		
**Country**					
United States	28		52,8		
Indonesia	1		1,9		
India	7		13,2		
United Kingdom	6		11,3		
I taly	3		5,7		
Canada	5		9,4		
Turkey	3		5,7		
**Video Content**					
Experience	14		26,4		
Educational	38		71,7		
Misleading Information	1		1,9		

The data are presented as mean ± standard deviation, median (minimum–maximum), and n (%).

It was found that videos uploaded by dentists had significantly higher median VCQ scores, GQS scores and DISCERN scores compared to those uploaded by individual users and advertisers. When examined by video content, educational videos had significantly higher VCQ scores, GQS scores, and DISCERN scores (*P* < 0.001). A significant correlation was found between video duration and VCQ score (rs = 0.83, *P* < 0.001). As the video duration increased, the VCQ score also tended to increase. Similarly, there was a significant correlation between video duration and GQS score (rs = 0.72, *P* < 0.001), indicating that longer videos were associated with higher GQS scores. A significant relationship was also observed between video duration and DISCERN score (rs = 0.69, *P* < 0.001), with DISCERN scores increasing as video length increased (Table 2).

**Table 2:** Comparison of VCQ, GQS, and DISCERN Scores According to Video Source, Content, and Correlation with Video Duration.

	VCQ score	GQS score	DISCERN score
**Video Source**			
İndivudual user	7(2-9)	2(1-3)	2(0-3)
Dentist	11(8-23)	3,5(2-5)	3(2-5)
Advertisement	6,5(6-7)	2(2−2)	2(2−2)
*P value*	<0,001^a^	<0,001^a^	<0,001^a^
**Post hoc p values**			
Individual user -Dentist	**<0,001**	**<0,001**	**<0,001**
Individual user -Advertisement	**–**	**–**	**–**
Dentist – Advertisement	**0.002**	**0.009**	**0.019**
**Video Content**			
Experience (n = 14)	7(2-9)	2(1-3)	2(0-3)
Education (38)	11(6-23)	3(2-5)	3(2-5)
Misleading Information (n = 1)*	–	–	–
*P value*	**<0,001** ^**b**^	**<0,001** ^**b**^	**<0,001** ^**b**^
**Video Duration**			
r_s_	0,832	0,723	0,692
*P value*	**<0,001**	**<0,001**	**<0,001**

Data are presented as median (minimum–maximum).

ᵃ: Kruskal-Wallis test, ᵇ: Mann–Whitney U test

*: Not included in the statistical analysis due to insufficient data.

r_s_: Spearman correlation coefficient

The analysis revealed a strong and significant positive correlation between GQS and VCQ scores and GQS and DISCERN scores respectively (ρ_s_ = 0.89, ρ_s_ = 0.73, *P* < 0.001). This finding indicates that videos with higher GQS scores also tend to have higher VCQ scores and higher DISCERN scores (Table 3).

**Table 3:** Correlation of GQS scores with VCQ and DISCERN scores in YouTube™ videos.

	VCQ score	DISCERN score
**GQS score**		
p_s_	0,895	0,734
*P* value	**<0,001**	**<0,001**

rs: *Spearman correlation coefficient.*

The overall inter-observer agreement, measured by the weighted kappa coefficient, demonstrated a strong agreement (κ = 0.897, P < 0.001).

## Discussion

This study is the first to systematically evaluate the quality, reliability, and educational value of YouTube™ videos related to lip repositioning surgery. The present study demonstrated that videos uploaded by dental professionals exhibited significantly higher VCQ, GQS, and DISCERN scores compared to those uploaded by individual users and commercial sources. Additionally, longer video duration was positively correlated with higher content quality and educational value. These results highlight the variability in the quality of available online information and underscore the need for professionally created, evidence-based content to improve patient and student education.

In our study, it was observed that 64.2% of the videos were uploaded by dentists. Similarly, in a study conducted by Kovalski et al., it was reported that 75% of the videos were uploaded by dentists [14]. Likewise, in a study by Yağız et al., which evaluated YouTube™ videos on botulinum toxin use in gummy smile treatment, it was noted that 73.7% of the videos were created by healthcare professionals [15]. In another study, it was reported that 88 videos (93.6%) were uploaded by healthcare professionals [16].

Findings from the literature suggest that the source of YouTube™ videos related to dentistry plays a significant role in determining content quality. In line with previous studies, we found that videos uploaded by healthcare professionals and academic institutions had higher VCQ and DISCERN scores [17–20]. Consistent with these findings, our study also revealed that videos created by dentists had significantly higher VCQ, GQS, and DISCERN scores compared to those uploaded by individual users and for promotional purposes. This supports the idea that content produced by healthcare professionals tends to be more comprehensive, scientific, and evidence-based. On the other hand, videos uploaded by businesses were largely marketing-oriented and may have a commercial focus rather than being informative. These findings highlight that while YouTube™ has potential as an important platform for health education, a more active role by professional content creators is important to ensure the reliability and educational value of the content.

In previous YouTube™-based studies published in the literature, it has been observed that the majority of the videos originated from the United States [21,22]. Our findings are consistent with this, showing that 52.8% of the videos with identifiable origins were uploaded from the United States. The dominance of the U.S. in this area may be attributed to the fact that YouTube™ is a U.S. based social media platform [23]. In addition, the recognition of English as the global language in health communication likely contributes to the prominent role of countries such as the United States and the United Kingdom in producing digital health content.

Various studies have shown that the duration of YouTube™ videos also has a significant impact on content quality. Research has reported that videos rated as “excellent” tend to be longer in duration compared to others [24–27].Recently, D’Ambrosi et al. evaluated YouTube videos in two separate studies and consistently reported a positive correlation between video quality and duration [16,28]. Consistent with the literature, our study also found a significant correlation between video duration and VCQ, GQS, and DISCERN scores. The average duration of the videos in our study was 3.93 minutes, and the video with the highest VCQ score had a duration of 14.32 minutes. These findings suggest that as video length increases, the scope and depth of the information presented tend to improve. Therefore, video duration should be considered an important factor in the development of health education materials. However, it should also be noted that excessively long videos may have a negative impact on viewer engagement. For this reason, we excluded videos longer than 15 minutes from our study. Interestingly, the most viewed videos in our sample were generally those with a duration of 7 minutes or less. While shorter videos seem to attract more interest from viewers, videos containing detailed and academic content tend to be longer in duration. In a study by Delli et al., it was reported that videos considered useful by viewers had an average duration of approximately 7 minutes [29]. In conclusion, video duration should be strategically balanced to maintain viewer engagement. Future research could provide further guidance by examining the impact of videos of varying lengths on viewer interaction, helping to determine the optimal video duration for educational effectiveness.

Dental school students often utilize YouTube™ videos as visual educational tools to acquire information. Some researchers actively encourage the use of YouTube™, especially among medical students, as a complementary resource for learning [27]. In a study by AlSilwadi et al., the importance of social media in increasing the knowledge level of patients undergoing fixed orthodontic treatment was investigated, and platforms like YouTube™ were found to contribute positively to patient education [30]. However, the findings of our study revealed that out of the 150 videos retrieved when searching the term “lip repositioning”, 97 videos (64.6%) were irrelevant, duplicated, or lacked audio or visual content. This is consistent with the findings of Elkarmi et al. who reported a similar rate of 50%, and Sadry et al. who found a rate of 62.7%, closely aligning with our results [31,32]. These findings clearly demonstrate that even when patients or healthcare professionals use correct search terms, they often face challenges in accessing reliable information due to the presence of irrelevant, repetitive, or low-quality videos. This highlights the pressing need for improved content regulation and curation on platforms like YouTube™ to enhance the reliability and usability of medical information.

In our study, based on VCQ scores, the majority of the videos (56.60%) were found to have poor content quality. This finding is consistent with previous studies in the literature that concluded YouTube™ videos are often insufficient as reliable sources of medical information [8,33,34]. According to GQS scores, the overall quality of the videos was determined to be moderate. This result is in line with the findings of Turkmen et al., who evaluated the quality of YouTube™ videos on gummy smile and reported similar outcomes [35]. Despite concerns regarding information gaps and content quality, it is evident that patients will continue to use online platforms such as YouTube™ as sources of health information. In this context, it is of great importance that health professionals direct patients to reliable, up-to-date and scientifically based information.

In our study, videos with higher GQS scores were also found to have higher VCQ and DISCERN scores. This finding indicates that the overall quality of the videos is proportionally associated with their content quality and reliability. Similar results have also been reported in previous studies in the literature [36,37].

This study has several limitations. First, the results are dependent on the keyword used; different search terms may yield different outcomes. Additionally, due to the constantly evolving nature of YouTube™, the results may vary depending on the date and time of the search. YouTube’s search results can be personalized based on factors such as user profile, geographic location, and mode of access, which may lead to variations in the list of retrieved videos across different users. One limitation of this study is that it included only English-language videos, despite the fact that YouTube™ is extensively accessed in non-English-speaking regions. This language restriction may reduce the overall generalizability of the study’s conclusions. In this study, tools such as the DISCERN instrument, Video Content Quality (VCQ) score, and Global Quality Scale (GQS) were utilized to assess the reliability, validity, and overall quality of video content. Although these instruments have not been formally validated, they are widely adopted in studies evaluating online health information as a standard approach

YouTube™ is a widely used platform for accessing health information; however, many videos on lip repositioning surgery lack sufficient quality and reliability. Videos uploaded by dental professionals showed higher content and overall quality. Encouraging the production of accurate and evidence-based content by professionals is essential to improve the educational value of such platforms.

## Supporting information

S1 Table URLs of the YouTube™ videos included in the study. (XLSX)

S2 Table Descriptive data of videos analyzed for lip repositioning surgery, including video duration, interaction index, viewing rate, source, content type, and quality scores (GQS, VCQ, and DISCERN). (XLSX)
